# Estimating the value of an early childhood education nutrition program

**DOI:** 10.3389/fnut.2025.1613236

**Published:** 2025-11-04

**Authors:** Ruchira Mahashabde, Allen Smith, Taren Massey-Swindle, Julie M. Rutledge, Zhang Dong, Jacob T. Painter

**Affiliations:** ^1^Division of Pharmaceutical Evaluation and Policy, Department of Pharmacy Practice, University of Arkansas for Medical Sciences, Little Rock, AR, United States; ^2^Department of Family and Preventative Medicine and Developmental Nutrition in Pediatrics, University of Arkansas of Medical Sciences, Little Rock, AR, United States; ^3^School of Human Ecology, Louisiana Tech University, Ruston, LA, United States

**Keywords:** early childhood nutrition, nutrition program evaluation, cost-effectiveness, childhood health outcomes, health economics

## Abstract

**Objective:**

The objective of the study was to estimate the cost, effectiveness, and value of the Together, We Inspire Smart Eating (WISE) intervention compared to usual nutrition education (UNE) in Arkansas Head Start programs that are part of the Child and Adult Care Food Program (CACFP).

**Methods:**

A pre-post study design with non-randomized group assignment was employed to compare cost, body mass index (BMI), and fruit and vegetable intake (measured using the Food Frequency Questionnaire, FFQ) among children aged 3–5 years between September 2015 and April 2018.

**Results:**

Children at WISE sites showed a greater increase in FFQ scores (0.29 units, *p* < 0.01) compared to those at UNE sites (0.05 units, *p* = 0.15), with no significant BMI differences between the groups. The WISE intervention costs $2.16 per child per month, whereas the UNE allocates $3.52 per child per month. Overall, WISE was both more effective and less expensive 25% of the time, costing $0.26 per 1-unit increase in FFQ scores. The WISE intervention remained favorable even when sites were unwilling to pay for its implementation.

**Conclusion:**

WISE promoted healthier diets at a lower cost than UNE, as evidenced by improved FFQ scores. These findings provide insights for decision makers regarding the intervention’s cost, potential savings, and overall value.

## Introduction

Childhood obesity and malnutrition are major concerns in pediatric public health in the United States (US). From 2017 to 2020, the average prevalence of obesity was 19.7%, affecting 14.7 million children and adolescents aged 2–19 years, including 13% of children aged 2–5 years (1). Paradoxically, many low-income families face simultaneous challenges of obesity and “hidden hunger” (micronutrient deficiencies) (2, 3). While caloric intake may be sufficient, these diets often lack essential vitamins and minerals necessary for healthy growth and development. Thus, food consumption and dietary patterns established early in life can influence obesity during childhood and later in life (4, 5). Childhood obesity is associated with a greater risk of comorbidities, including cancer (6–8). Overweight preschool children are five times more likely to remain overweight or become obese in adulthood (9). This risk is compounded by the overconsumption of energy-dense foods and inadequate intake of micronutrient-rich foods—a pattern that often begins in early childhood settings (10). Improving diet by reducing the consumption of unhealthy foods (e.g., fatty cheese, sugary drinks/foods, processed/refined foods, and animal products) while increasing the intake of healthy foods (e.g., fruits/vegetables, whole foods, fish, nuts, and yogurt, as well as foods low in added sugars and unhealthy fats) reduces the risk of developing obesity (9). Therefore, efforts aimed at preventing obesity by influencing children’s diet and nutrition during early childhood are essential.

While a nutritious diet improves overall wellbeing, economic factors may limit access to healthy foods and negatively influence nutritional status. A review of 27 studies from 10 countries revealed that healthier food options cost, on average, $0.29 more per serving and $1.48 more per day than less nutritious alternatives (11). Elevated food prices can disproportionately affect vulnerable populations, especially children, by restricting their ability to maintain a healthy diet (12). These economic barriers highlight the need to consider cost when evaluating nutrition-focused obesity prevention interventions.

Given the need to address obesity and malnutrition at an early age, the Early Care and Education (ECE) setting presents a promising intervention target. Approximately 60% of children under the age of five in the US spend 36 h per week in ECE settings, where they receive 30–50% of their daily calories through school meals (13, 14). These settings are critical for breaking cycles of both overeating and micronutrient deficiencies through structured meals and nutrition education. Programs such as the Child and Adult Care Food Program (CACFP) fund many of these meals and snacks, with eligible Head Start programs receiving reimbursements for these provisions from the CACFP. Understanding the cost associated with these interventions is essential for education site leaders as they make decisions that involve complex budget planning (15).

Together, We Inspire Smart Eating (WISE) is an evidence-based, direct nutrition education program designed to improve children’s diets in ECE settings (16). This program encourages children to eat carotenoid-rich fruits and vegetables while fostering healthy growth and self-regulated eating behaviors (16). Sites that implemented the WISE intervention have shown a significantly greater increase in fruit and vegetable consumption compared to sites receiving usual nutrition education (UNE), along with an 8% rise in skin carotenoid biomarkers, indicating a higher intake of carotenoid-rich fruits and vegetables (17) In addition, parent reports indicated a decrease in children’s consumption of fast food and sugar-sweetened beverages after 1 year of participating in the WISE intervention (18).

Although programs such as WISE are often regarded as valuable due to their positive outcomes, there is an increasing focus on evaluating their economic return. High-impact programs that come with significant costs may not always be the most effective option; therefore, this study seeks to assess the cost of implementing WISE and its incremental cost-effectiveness ratio (ICER) compared to UNE in Head Start programs in Arkansas, United States, which receive reimbursements for snacks from the CACFP. The findings will help decision makers understand the cost associated with WISE, identify potential cost offsets, and provide an estimate of its overall value in addressing obesity and malnutrition through early childhood nutrition programs.

## Methods

### Study design

The study used a quasi-experimental, pre-post design with a non-randomized control group to evaluate the cost-effectiveness of WISE compared to UNE. The analysis used the perspective of a single Head Start site, as site-level leadership typically decides which interventions to adopt. The study included 23 Head Start sites in Arkansas—6 of which received the WISE intervention and 17 of which received the UNE intervention. Children aged 3–5 years enrolled at these Head Start sites were included as participants. Costs and outcomes related to the intervention were measured between September 2015 and April 2018.

### Intervention

Details of the intervention have been described in a previous publication (19). In summary, WISE includes weekly classroom lessons, educator training, and family engagement resources, and a puppet named “Windy Wise” encourages healthy habits in the classroom. WISE focuses on eight fruits and vegetables throughout the school year, with one highlighted each month.

### Study measures

The study evaluated effectiveness using two primary outcomes: a composite score from a modified version of the Food Frequency Questionnaire (FFQ) and body mass index (BMI) compared to the 95th percentile. Intervention-related costs included expenses for food and necessary supplies. FFQ scores and BMI percentile data were collected before and after the intervention was implemented at each site. WISE intervention costs were calculated on a per-child, per-month basis. Children in the WISE group received the intervention over the course of an academic year (2013–2014 or 2014–2015).

Each child’s BMI was measured at both WISE and UNE sites as part of routine Head Start procedures. At WISE sites during the 2013–2014 academic year, BMI data collected between September and October 2013 were designated as pre-intervention, while those recorded from March to April 2014 were considered post-intervention. Similarly, at WISE sites during the 2014–2015 academic year and at all UNE sites, BMI data collected between September and October 2014 were designated as pre-intervention, while data from March to April 2015 were classified as post-intervention. According to CDC guidelines, a child’s BMI should be expressed as a percentile in comparison to peers of the same sex and age in the US (20). Children with BMI values at or above the 95th percentile are considered obese (21). Following this recommendation, all BMI data in this study were calculated relative to the 95th percentile. These BMI percentile scores were averaged at the site level for analysis. It was hypothesized that BMI reductions would be greater at WISE sites compared to UNE sites over the course of the study.

A modified version of the FFQ was used to focus on foods targeted by the WISE intervention (22–24). The FFQ is a validated instrument for estimating a child’s intake of fruits, vegetables, and less nutritious foods. A parent or guardian is responsible for completing the questionnaire. The development and validation of the questionnaire have been detailed in a previous publication (18). In a modified approach, Head Start conducted interviews with caregivers before and after WISE activities, typically during the summer before the fall implementation and again in the spring before the school year ended. From these responses, the average monthly consumption of WISE-targeted fruits and vegetables for each child was calculated. These individual scores were then averaged across each site for use in the analysis.

#### Cost assessment

Study invoices were used to gather data on expenditures for food, materials, and training at WISE sites. From these data, the average monthly cost per-child for implementing the WISE intervention at each site was calculated. The CACFP snack reimbursement rate served as a comparator for UNE sites. This rate reflects a national average and is adjusted annually in July based on the Consumer Price Index (CPI-U). For this analysis, the reimbursement rate of $3.52 for the 2017–2018 CACFP year was applied (25). Costs incurred at WISE sites before 2018 were adjusted for inflation to reflect 2018 dollar values using the CPI-U data from the US Department of Labor Bureau of Labor Statistics.

### Statistical analysis

Analyses were conducted using both individual child data and site-level aggregates. Although measurements were collected at the individual child level, we analyzed and interpreted the results at the site level for two key reasons: (1) the cross-sectional and unpaired nature of the measurements due to varying child attendance patterns before and after the intervention, and (2) the policy relevance of understanding the program effectiveness at the implementation level for decision makers. For cost calculation at WISE sites, average outcome costs were calculated at the site level across all years and then weighted by the number of children who received the intervention at each WISE site to determine the WISE cost per child per month. Weighted averages and standard deviations for BMI and the FFQ were calculated for each study group. Independent samples *t*-tests were performed to compare pre- and post-intervention effectiveness measures for children at WISE and UNE sites separately. In the cost-effectiveness analysis (CEA), which evaluates the relative value of health programs, an incremental cost-effectiveness ratio (ICER) was calculated to assess whether the value provided by WISE justified its implementation cost. The ICER represents the ratio of the difference in cost and effectiveness between two interventions. If an intervention is both more costly and more effective than its alternative, the ICER helps decision makers assess whether the added benefits are worth the extra cost. For this analysis, the ICER is expressed as dollars per unit reduction in BMI and dollars per unit increase in the FFQ score. We assumed that WISE intervention costs were normally distributed across sites.

Since the proportion of the CACFP reimbursement allocated to UNE-related expenses is unknown, a uniform distribution between $0 and $3.52 was assumed. A total of 1,000 random incremental costs and effectiveness predictions were generated using these assumed distributions. Using these predictions, a graph showing the joint distribution of costs and effectiveness was constructed, known as the cost-effectiveness analysis (CEA) plane. The CEA plane shows differences in effectiveness between WISE and UNE on the horizontal axis and differences in cost on the vertical axis. Higher effectiveness of WISE appears farther to the right on the CEA plane. Higher costs for WISE appear higher on the CEA plane. A cost-effectiveness acceptability curve (CEAC) was also created to show the probability that WISE is cost-effective compared to UNE across a range of willingness-to-pay (WTP) thresholds that ECE decision makers might accept for a one-unit increase in the FFQ score or one-unit decrease in BMI. All analyses were conducted using Microsoft Excel and SAS version 9.4 (SAS Institute, Cary, NC, United States). The significance level was set at 0.05 for all statistical tests.

## Results

On average, the FFQ score increased by 0.29 units among children at WISE sites (*t*-value = 7.06; *p* < 0.01), while children at UNE sites experienced only a 0.05-unit increase (*t*-value = 1.43; *p* = 0.15). No significant differences in BMI were found between WISE and UNE sites. Table 1 presents the monthly and annual per-child costs of implementing WISE. The average monthly cost of the WISE intervention per child was $2.16. This amount represents approximately 60% of the $3.52 CACFP reimbursement received by the sites.

**Table 1:** Inflation adjusted amounts spent during WISE.

Year	Months	Number of children who received the WISE intervention	Inflation adjusted amount for WISE food per year	Inflation adjusted amount spent during WISE per child per month
2015–2016	Sept-Apr	240	$ 3,245.88	$ 2.10
2016–2017	Sept-Apr	240	$ 2,898.27	$ 1.83
2017–2018	Sept-Oct	240	$ 478.25	$ 2.40
	Nov-Dec	40	$ 331.95	$ 10.72
	Jan-Apr	303	$ 2,047.83	$ 7.40
From 2015 September to 2018 December the average cost of WISE intervention per child per month is				$ 2.16

**Figure 1:**
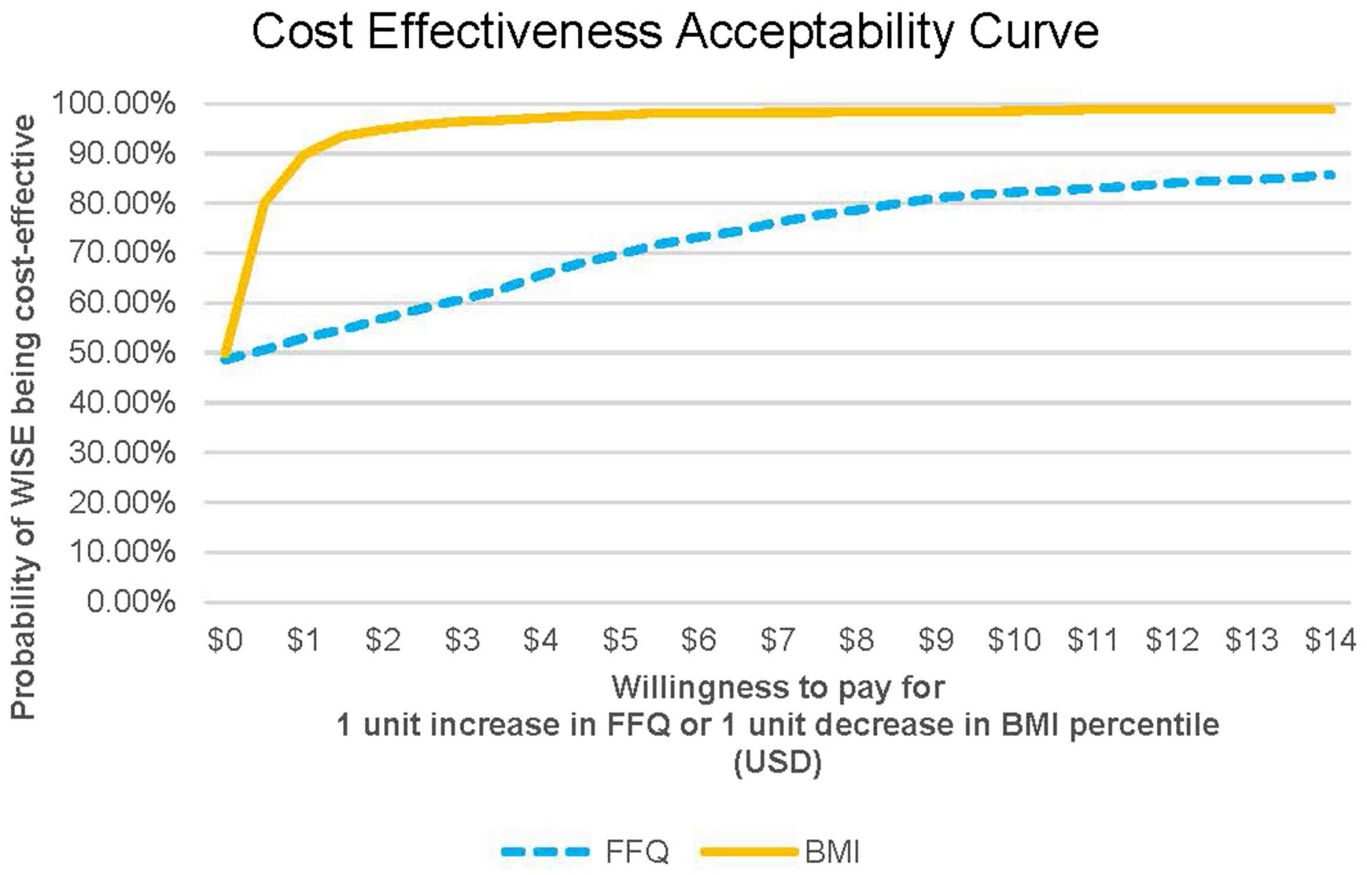
Cost effectiveness analysis curve for FFQ and BMI. FFQ, food frequency questionnaire outcome; BMI, BMI relative to 95th percentile outcome.

**Figure 2:**
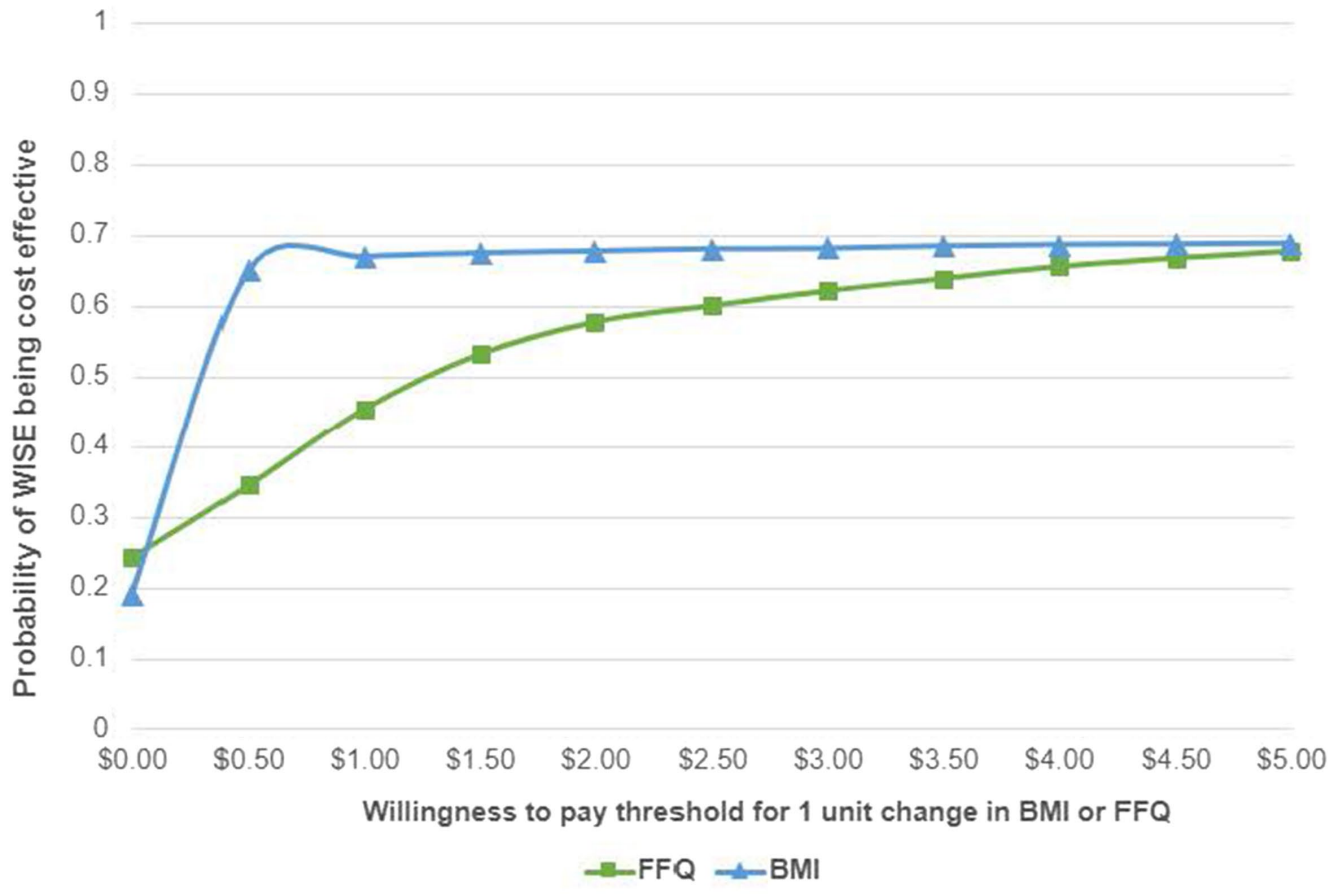
Cost effectiveness acceptability curve for FFQ and BMI. FFQ, food frequency questionnaire outcome; BMI, BMI relative to 95th percentile outcome.

Figure 1 shows the CEA plane for the FFQ, where 36% of predictions placed WISE in the northeast quadrant, indicating that it was both more costly and more effective than UNE in those instances. WISE was considered dominant (i.e., more effective and less costly) in 25% of the predictions. The average ICER was $0.26 per child per month per additional FFQ unit, indicating that it costs approximately $0.26 more per child per month at WISE sites to achieve a one-unit increase in the FFQ score compared to UNE sites. No significant effects were observed for BMI outcomes. Figure 2 shows that when the WTP is $0, WISE remains the preferred option over UNE approximately 50% of the time.

## Discussion

This study evaluated the cost-effectiveness of WISE, an evidence-based nutrition education program implemented in the ECE setting. Our findings demonstrated a significant increase in the intake of carotenoid-rich fruits and vegetables among children at WISE sites after the intervention. In addition, Wise program costs ($2.16) were lower than the allowed CACFP reimbursement rate ($3.52) per child per month. However, no significant changes in BMI levels were found among children at both WISE and UNE sites. The program’s ability to enhance diet quality at lower costs suggests strong potential for implementation in resource limited settings at a reimbursable cost.

The significant improvement in FFQ scores among children at WISE sites compared to UNE sites (0.29 vs. 0.05 units, *p* < 0.01) confirms the intervention’s effectiveness. This finding aligns with and extends earlier research demonstrating that WISE improved fruit and vegetable consumption among low-income preschool and early elementary students in Arkansas (17). These findings further align with broader evidence on ECE-based nutrition programs, which have been shown to improve fruit/vegetable intake by approximately 0.08 to 0.14 servings/day, according to a meta-analysis of healthy eating interventions (26). Notably, FFQ scores specifically capture the consumption of nutrient-dense, carotenoid-rich foods (e.g., sweet potatoes, carrots, leafy greens), which are particularly important for addressing micronutrient deficiencies common in low-income populations (27). These results suggest that programs such as WISE can possibly help improve nutrition among young children in low-income communities and may be a practical way to promote healthier eating habits in early life.

While WISE significantly improved fruit and vegetable intake, no changes in the BMI of children were detected. This is consistent with evidence that pediatric lifestyle or policy interventions generally require longer timelines (12–24 months) to show clinically meaningful BMI changes, whereas dietary behaviors may shift rapidly (27, 28). Given that our study truly focused on cost-effective dietary improvement rather than caloric restriction, it is unsurprising that BMI differences were not detected. For policymakers prioritizing short-term improvements in diet quality, WISE offers measurable value. However, extended studies with broader timelines are required to determine whether sustained WISE participation translates to BMI changes.

In our analysis, 36% of the ICER predictions were in the northeast quadrant of the CEA plane, indicating that WISE was both more costly and more effective than UNE in those cases. Determining value from ICERs requires comparing them to a defined monetary WTP threshold, representing the maximum a decision maker is willing to pay for additional effectiveness (29). We used the full $3.52 CACFP reimbursement rate as a conservative proxy for the maximum WTP for the WISE intervention, although only part of this amount typically goes toward direct food purchases. However, even with a WTP of $0, WISE had roughly a 50% probability of being cost-effective, with this probability increasing as the WTP rises.

With its combination of improved dietary outcomes and costs well below the CACFP reimbursement threshold, WISE presents actionable pathways for systemic change. First, policymakers could incentivize adoption by permitting a portion of CACFP funds to support evidence-based nutrition education, a strategy that has proven successful in SNAP-Ed programs (30). In addition, WISE has approximately a 50% chance of being cost-effective even when the WTP is $0, making it a realistic option for programs with limited budgets. These strategies could expand WISE’s reach without needing new funding, which is especially important in today’s tight budget climate.

There are several limitations that should be acknowledged. First, while the modified FFQ was administered by the staff using standardized protocols to minimize variability in data collection, BMI measurements were based on routine operational practices. This resulted in different children being measured at the beginning and end of the intervention based on attendance. Consequently, BMI outcomes represent site-level, cross-sectional trends rather than individual-level changes and should be interpreted accordingly. Second, because it is unclear how much of the CACFP reimbursement is allocated to direct food purchases, we modeled the WTP using a uniform distribution ranging between $0 and $3.52. Comparisons between the full $3.52 CACFP reimbursement and the $2.16 WISE cost should be made cautiously, as CACFP funds also cover expenses other than food purchasing. Third, our analysis did not include covariate adjustment, which may increase the risk of selection bias if the baseline characteristics differed between the children in the intervention (WISE) and control (UNE) groups. Due to the real-world nature of this study, the variables collected were limited to cost-effectiveness metrics relevant to policymakers. Although our unadjusted analysis lacks causal inference, it provides practical estimates of WISE’s costs and benefits, which are key considerations for resource allocation in ECE settings. Fourth, due to the unpaired nature of the data, we presented the estimates as site-level aggregates. Although this approach provides meaningful evidence for decision makers, it may obscure some individual-level variation in intervention effects. Fifth, UNE’s cost estimates relied on reimbursement rates and were assumed, rather than observed, expenditures. Future studies could collect detailed cost accounting data at the Head Start level to improve accuracy. These findings are exploratory and should be interpreted as associations rather than causal effects.

## Conclusion

Implementing WISE costs $2.16 per child per month, which is approximately 60% of the $3.52 CACFP snack reimbursement received by UNE sites. In addition, WISE sites showed greater FFQ score improvements than UNE sites. There is limited research on the cost-effectiveness of nutrition education programs in early childhood settings. However, our findings suggest that WISE would be considered a valuable investment by decision makers—even at minimal WTP levels. Future research should explore WTP thresholds in ECE settings and use adjusted models with longer follow-up periods to better assess causal effects. For practice, our results suggest that low-cost, nutrition-focused programs such as WISE can improve dietary quality at scale, supporting more equitable access to healthy foods in early childhood settings.

## Data Availability

Data are available upon reasonable request by contacting the corresponding author.
